# Spatio-Temporal Dynamic of *Tuber magnatum* Mycelium in Natural Truffle Grounds

**DOI:** 10.1371/journal.pone.0115921

**Published:** 2014-12-23

**Authors:** Mirco Iotti, Marco Leonardi, Enrico Lancellotti, Elena Salerni, Marilena Oddis, Pamela Leonardi, Claudia Perini, Giovanni Pacioni, Alessandra Zambonelli

**Affiliations:** 1 Department of Agricultural Sciences, Bologna University, Bologna, Italy; 2 Department of Life, Health and Environmental Sciences, L'Aquila University, L'Aquila, Italy; 3 Dipartimento di Agraria, Sassari University, Sassari, Italy; 4 Department of Life Science, Siena University, Siena, Italy; DOE Pacific Northwest National Laboratory, United States of America

## Abstract

*Tuber magnatum* produces the world's most expensive truffle. This fungus produces very rare ectomycorrhizas which are difficult or even impossible to detect in the field. A “real-time” PCR assay was recently developed to quantify and to track *T. magnatum* mycelium in soil. Here, this technique was used to investigate the spatial distribution of *T. magnatum* extra-radical mycelium in soil productive patches and its dynamic across seasons. This study was carried out in four different natural *T. magnatum* truffle grounds located in different Italian regions. During the fruiting seasons, the amount of *T. magnatum* mycelium was significantly higher around the fruiting points and decreased going farther away from them. Moreover, *T. magnatum* mycelium inside the productive patches underwent seasonal fluctuations. In early spring, the amount of *T. magnatum* mycelium was significantly higher than in summer. In summer, probably due to the hot and dry season, *T. magnatum* mycelium significantly decreased, whereas in autumn it increased again and was concentrated at the putative fruiting points. These results give new insights on *T. magnatum* ecology and are useful to plan the most appropriate sampling strategy for evaluating the management of a truffle ground.

## Introduction

*Tuber magnatum* Pico is an ectomycorrhizal (ECM) ascomycete producing edible hypogeous ascomata (the Italian white truffle) which are one of the world's most expensive foods [1]. In addition to the valuable culinary properties, the high prices commanded for its ascomata reflect their low availability on the market. In Autumn 2012, a year characterized by poor harvests, *T. magnatum* was sold for 4000–5300 € kg^−1^ (http://www.tuber.it/pagine/ita/la_borsa/la_borsa.lasso). Attempts to cultivate *T. magnatum* have often failed and the global production is restricted to specific habitats that are scattered through the Italian and Balkan peninsulas [1]. In contrast, the other precious truffle species have been successfully cultivated around the world. *Tuber melanosporum* Vittad., *Tuber borchii* Vittad. and *Tuber aestivum* Vittad. productive orchards are located both in the Northern and Southern Hemispheres [2]–[4] and plantations of the desert truffle *Terfezia clavery* Chatin have been established in Mediterranean environments and in arid or semiarid soils of other countries [5]. The failure of *T. magnatum* cultivation is due to the poor scientific knowledge gathered for this truffle during the past few decades [6]. In fact, for *T. magnatum*, it has not been possible to apply the traditional experimental strategies adopted to study biology and ecology of the other *Tuber* species. The difficulties in synthesizing and maintaining its mycorrhizas in controlled conditions (axenic and greenhouse) [6]–[7] prevented the possibility to optimise conditions for root colonization as well as to study plant-fungus molecular interactions as for *T. borchii* [8]. Also, the scarcity of *T. magnatum* mycorrhizas in the field [9]–[10] has significantly hindered understanding of its spatial distribution pattern in soil and the effects of biotic and abiotic factors. Till now, field trials have only been aimed at describing environmental habitats and characterizing ECM communities where *T. magnatum* grows [10]–[13].

Pure cultures of *T. magnatum* have been recently obtained, but the *in vitro* growth of its mycelium is very poor and does not produce adequate amount of biomass for experimental purposes [14]. In contrast, a *T. magnatum* mycelial network in soil has been found to be more widespread than can be inferred from the distribution of its ascomata and ectomycorrhizas and this suggests that a study of it may help unravel the ecology of this truffle [15].

Large scale application of basic and advanced molecular methods in experimental microbiology has greatly improved the knowledge of the distribution of soil-inhabiting fungi, functioning and dynamics. Mycelium of a fungal species can be efficiently estimated by denaturing gradient gel electrophoresis (DGGE), cloning techniques, fluorescence in situ hybridization (FISH), real-time PCR (qPCR) and, more recently, next generation sequencing platforms [16]–[19]. Among these, qPCR proved to be a robust, highly reproducible and sensitive tool to track phylogenetic marker and functional genes present within environmental samples across temporal and spatial scales [20]. This molecular technique has been optimised to quantify ECM mycelium of several ECM fungi [16], [21]–[24] including the truffle species *T. melanosporum* and *T. aestivum* [25]–[27].

Recently, a qPCR assay has been also developed to quantify *T. magnatum* in soil [28] and it was also successfully applied to verify the effects of soil tillage on its mycelium [29]. These studies were carried out on a set of plots established in different truffle grounds to verify the relationships between *T. magnatum* mycelium, fruiting body production and soil disturbance. However, no information was provided on the distribution and dynamic of *T. magnatum* mycelium within the soil patches where this truffle species fructify. In this study we aimed to increase the knowledge on *T. magnatum* development in soil by assessing the spatial and temporal distribution of its mycelium in productive patches using the qPCR assay previously developed by Iotti *et al.* [28]. It also represents the first attempt to determine whether and how soil, habitat and seasonal climate conditions affect the *T. magnatum* mycelium biomass in soil.

## Materials and Methods

### Study sites

Research was carried out in four natural truffle grounds located along 400 km from North to South of the Italian peninsula and representative of productive *T. magnatum* areas (S1 Fig.). Study sites differ largely in elevation, soil type, climate, vegetation composition, human-induced disturbance and ECM fungal communities [10]. The northern site is inside the park of the “Bonifica Renana” Museum at Argenta (Ferrara, Emilia-Romagna) (latitude 44° 37′ 10″ N, longitude 11° 48′ 55″ E, altitude 5 m asl), located in a former swampy area of the Po river valley that were cleared and drained in ancient times for agriculture. The ground is nearly flat, covered by ECM host and non host plants as well as a number of exotic tree species (S1 Table). The soil is sandy loam, calcareous and moderately alkaline (Table 1).

**Table 1:** Soil characteristics of the four experimental sites.

Locality (Region)	Texture (%)			pH	AC (%)	BD	OM (%)
	Sand	Silt	Clay				
**Argenta (Emilia-Romagna)**	58.8	31.4	9.8	8.2	3.20	1.18	4.44
**Barbialla (Tuscany)**	60.2	29.9	9.9	8.2	0.73	1.17	3.53
**Feudozzo (Abruzzo)**	40.5	36.0	23.5	7.3	1.34	1.21	4.31
**Collemeluccio (Molise)**	41.6	26.4	32.0	7.3	2.55	1.20	3.34

Data refer to the top 30 cm of soil. AC, active carbonate; BD, bulk density; OM, organic matter.

The other three experimental sites are semi-natural woodlands in the Apennine mountains of central-southern Italy. The truffle ground of central Italy is a mixed forest inside the “Barbialla Nuova” private farm at Montaione (Florence, Tuscany) (43° 35′ 36″N, 10° 51′ 41″ E, altitude 135 m asl). Woody vegetation is almost entirely composed of deciduous broad-leaved species (S1 Table) and its management is limited to the removal of dead and hazardous trees. The ground slope ranges between 5% and 25%, whereas the soil is sandy loam and moderately alkaline (Table 1).

The southern sites are located in two reserves managed by the Biodiversity Office of the State Forest Service: “FDR Torre di Feudozzo” (Castel di Sangro, L'Aquila, Abruzzo) (41° 45′ 55″ 80 N, 14° 11′ 12″ 80 E, altitude 950 m asl), and “Riserva M&B di Collemeluccio” (Pescolanciano, Isernia, Molise) (41° 42′ 07″ 60 N, 14° 20′ 34″50 E, altitude 810 m asl). These truffle grounds are former coppice-with-standards forests which were converted to high forest of *Quercus cerris* L. and other hardwood species (S1 Table). A number of silver firs (*Abies alba* Miller), where all *T. magnatum* ascomata harvests occurred, were also present at Collemeluccio (S1 Table). Both experimental stands have a slight slope (0–4%) with clay loam (Collemeluccio) or loam (Feudozzo) and neutral or slightly alkaline soils (Table 1).

Detailed descriptions of climate and vegetation of each experimental truffle ground are provided in Table 2 and S1 Table, respectively. The following public and private authorities granted permission for the fieldwork and soil sampling: the board chairman of the “Bonifica Renana” consortium (Argenta experimental site), the owner of the “Barbialla Nuova” private farm (Montaione), and the managing directors of Isernia and Castel di Sangro Offices for Biodiversity of State Forest Service (Feudozzo and Collemeluccio). The field sampling did not involve endangered or protected species.

**Table 2:** Climate data for the studied truffle ground over the 3-year survey (2008–2010).

Climatic parameters	Barbialla			Argenta			Feudozzo		
	2008	2009	2010	2008	2009	2010	2008	2009	2010
**Mean annual T**	15.9	16.2	14.9	14.3	13.2	12.2	11.6	11.7	12.3
**Annual P**	827	655	741	598	485	670	908	1154	891
**Mean T hottest month**	25.4	27.4	26.1	25.1	23.6	25.9	20.5	20.7	20.6
**Mean max T hottest month**	32.6	34.7	32.3	32.1	36.5	38.2	29.4	28.6	27.9
**N° rainy days**	123	94	100	73	77	-	179	168	201

Collemeluccio data are not reported since no meteorological station is present in this locality but it is about 20 km far from Feudozzo. T =  temperature (°C); P =  precipitation (mm); - =  no data available.

### Truffle production and soil sampling

Whole truffle production from each site under investigation was assessed by trained dogs during weekly surveys from mid September to late December of three consecutive years (2008–2010). All *T. magnatum* ascomata collected were weighed and their position recorded.

A sampling strategy targeted at the productive patches was adopted to study *T. magnatum* mycelium in soil. Consequently, a variable number of ascomata per year and truffle ground were selected for sampling the soil around it, depending upon the amount and distribution of seasonal truffle production (Table 3).

**Table 3:** Number of productive spots sampled in each experimental site for spatial and seasonal analyses of *T. magnatum* extra-radical mycelium in soil.

Truffle ground	2008	2009			2010		
	Aut^a^	April	July	Aut^†^	April	July	Aut^a^
**Spatial analysis**							
**Argenta**	7 (8)			2 (2)			5 (5)
**Feudozzo**	2 (2)			7 (11)			3 (11)
**Barbialla**	9 (10)			3 (3)			4 (5)
**Collemeluccio**	2 (2)			3 (9)			2 (6)
**Temporal analysis**							
**Argenta**	7	7	7	7^b^			
**Feudozzo**				7	7	7	7^b^

a total number of *T. magnatum* ascomata collected in each site during the fruiting season (Sep–Dec) is given between brackets.

b Sampling carried out in November.

Spatial distribution of extra-radical mycelium was assessed in all experimental sites by processing soil samples collected during three *T. magnatum* seasons (2008–2010), from September to December. Three samples were obtained from the area around each selected ascoma (productive spot): one in the immediate surroundings (<5 cm) of the fruiting point (P0) and the other two at 100 cm (P1) and 200 cm (P2) from it. Each sample was composed of four soil cores (30 cm in depth and 1.6 cm in diameter) taken along the cardinal directions through the fruiting point by using disposable polyvinyl chloride tubes. Sampling was carried out within 24 hours from the collection date of each ascoma. A total of 147 soil samples (49 productive spots, 3 distances) were processed for studying spatial distribution of *T. magnatum* extra-radical mycelium (Table 3).

The seasonal dynamic of *T. magnatum* mycelium was also assessed in Argenta and Feudozzo truffle grounds. Seven productive spots for each site, previously sampled in Autumn for studying spatial distribution of mycelium, were also sampled in the following April, July and November 2009 (Argenta) or 2010 (Feudozzo), adopting the same approach as described above. Samplings were performed in different years in order to analyse an adequate number of productive spots through the seasons. A total of 126 soil samples (7 productive spots, 2 truffle grounds, 3 distances, 3 seasons) were processed for evaluating seasonal dynamic of *T. magnatum* mycelium.

Soil cores taken at the same time and distance from each fruiting point were pooled together and any root fragment, stone or organic debris was carefully removed under a stereomicroscope (8 x). Soil samples were freeze-dried at −60°C for three days in a Virtis Benchtop 2 K lyophilizer (SP Industries) and then pulverized by mortar and pestle and finely homogenized. Fifty to 180 g of dried soil was recovered for each sample depending on soil characteristics. Three 15 ml-tubes containing 5 g of soil were prepared for each sample and then stored at −20°C until DNA extraction.

### DNA extraction and qPCR assay

DNAs were isolated using a CTAB-based buffer (2% CTAB, 2% Polyvinylpyrrolidon, 2 M NaCl, 20 mM EDTA, 100 mM Tris–HCl, pH 8), following the protocol described by Iotti *et al.* [28]. Crude DNA solutions were then purified using the Nucleospin Plant II kit (Macherey-Nagel). Total DNAs were quantified by a NanoDrop ND-1000 Spectrophotometer (Thermo Scientific) and their quality evaluated with optical density (OD) 260/280 nm and 260/230 nm ratios. Extractions with OD ratios or DNA concentration lower than 1.4 and 25 ng µl^−1^, respectively, were repeated. DNA solutions were kept at −20°C until processing.

Quantification of *T. magnatum* DNA in soil samples was performed by qPCR using the specific primer pair and TaqMan probe proposed by Iotti *et al.* [28]. DNA extracts were amplified in 96-well optical plates (Bioplastic) using a Stratagene Mx3000P QPCR system (Stratagene). Twenty five µl reaction volumes containing 12.5 µl (1×) of Maxima Probe qPCR Master mix (Fermentas), 30 nM of ROX, 0.5 µM of each primer and 0.2 µM of TaqMan probe (5′-6-FAM reporter dye, 3′-TAMRA quencher dye) (MWG BIOTECH) were prepared in duplicate for each soil DNA extract, negative control and standard. Two hundred nanograms of total DNA were added to each reaction. In order to make the comparison of the data from different runs as reliable as possible [30], identical standard curves were prepared for each plate at the beginning of the experiment from a single series of ten-fold dilutions of *T. magnatum* genomic DNA (from 10^7^ to 10^2^ fg per reaction) as standards. Moreover, samples from the same fruiting spots were processed in the same plate. Mean standard curve generated from 28 independent qPCR runs is shown in S2 Fig..

QPCR cycling conditions were 10 min at 95°C followed by 40 cycles of 95°C for 15 s, 60°C for 30 s and 72°C for 30 s. For each run, cycle threshold (Ct) values were automatically calculated and converted to quantities of *T. magnatum* DNA by the MXPro software (version 4.10) (Agilent technologies). qPCRs were repeated twice to confirm the results.

### Quantification of *T. magnatum* mycelial biomass

A soil-specific calibration curve was generated for each truffle ground to convert DNA concentrations obtained from qPCRs to absolute quantities of *T. magnatum* mycelium and, so, to compare the results obtained from the different soil types. For this purpose, known amounts of a completely immature ascoma (without spores) were added to soils free from *T. magnatum* mycelium. Ascoma was selected over mycelium because of the inability to grow suitable pure cultures of *T. magnatum* [14], whereas the soils were obtained from a number of samples collected in unproductive truffle patches of each experimental site. Ascoma tissue (gleba) and soil samples were lyophilised and ground as described above. In order to insure the effective absence of *T. magnatum* mycelium in soil samples candidate to generate the calibration curves, total DNAs were preliminarily isolated by aliquots of these samples using the Nucleospin Soil kit (Thermo Scientific) and then amplified with the specific primer pair and conditions mentioned above. Soil samples from the same truffle ground not generating PCR products were pooled together and split in 24 sub-samples of 5 g for DNA extraction. Tenfold serial dilutions of a 50 mg ml^−1^ suspension of gleba powder in CTAB lysis buffer were then prepared and 250 µl of each dilution were added to the soil sub-samples free from *T. magnatum* mycelium. A total of 7 serial dilutions of *T. magnatum* gleba per g of soil (from 2 mg to 2*10^−6^ mg) were processed in triplicate for each truffle ground. *T. magnatum* mycelium-free soil samples were used as negative controls. DNA extractions and qPCRs were performed as described in the previous subheading. A total of 48 qPCR reactions were performed to generate each calibration curve [8 samples (7 serial dilutions and the negative control) ×3 biological replicates ×2 technical replicates).

Calibration curves were generated by plotting the pg of *T. magnatum* DNA obtained by the standard curve versus the mg mycelium added per g of soil from each experimental site. Quantities of *T. magnatum* mycelial biomass in soil samples collected at different times and distances were determined by interpolation of their DNA value into the calibration curve generated for the corresponding truffle ground.

### Statistical analyses

Two different datasets were generated and analyzed to explain the spatial distribution and temporal variation of *T. magnatum* mycelium in soil. A stepwise procedure based on the Bayesian Information Criterion (BIC) [31] was used to verify whether other variables had to be retained in the statistical models in addition to sampling position and sampling season (target variables). Stepwise procedure is a variable selection method of regression (backward/forward direction) which allows to eliminate non-relevant variables from the model and to simplify the interpretation of statistical results. The set of variables considered for spatial distribution analysis of *T. magnatum* mycelium was composed by sampling position (P0, P1, P2), truffle ground (Barbialla, Argenta, Collemeluccio, Feudozzo), year of sampling (2008, 2009, 2010), and their interactions. Variables considered for studying mycelium dynamics in Argenta and Feudozzo truffle grounds were sampling season [fruiting time (September–December), April, July, November], sampling position and their interaction. Differences in *T. magnatum* mycelial biomass for the selected variables were analysed by ANOVA with Tukey's honestly significant difference (HSD) used as a test to separate means. Data were log-transformed after Bartlett's test to meet the ANOVA requirements of homogeneity of variance. The datasets used for statistical analyses are shown in S1 File.

The correlation between weight of *T. magnatum* ascomata and soil mycelial concentration in the fruiting points (P0) was assessed by Pearson correlation coefficient. All analyses were conducted with R (version 2.14.12) [32].

## Results

A total of 819 DNA extracts (273 soil samples per 3 biological replicates) were processed from the four truffle grounds surveyed for the three years of experimentation. A mean amount of 6.24 µg of isolated total DNA per g of soil with OD_260/280 nm_ and OD_260/230 nm_ ratios of 1.75 and 1.70, respectively, was obtained. Significant differences in DNA yields were observed among the soil samples from Collemeluccio and Feudozzo (lowest values) and Argenta and Barbialla (highest values) truffle grounds (*p*<0.0001). On the contrary, no statistical differences in DNA yields were found among the soils collected from the different sampling positions (*p* = 0.65).

**Figure 1:**
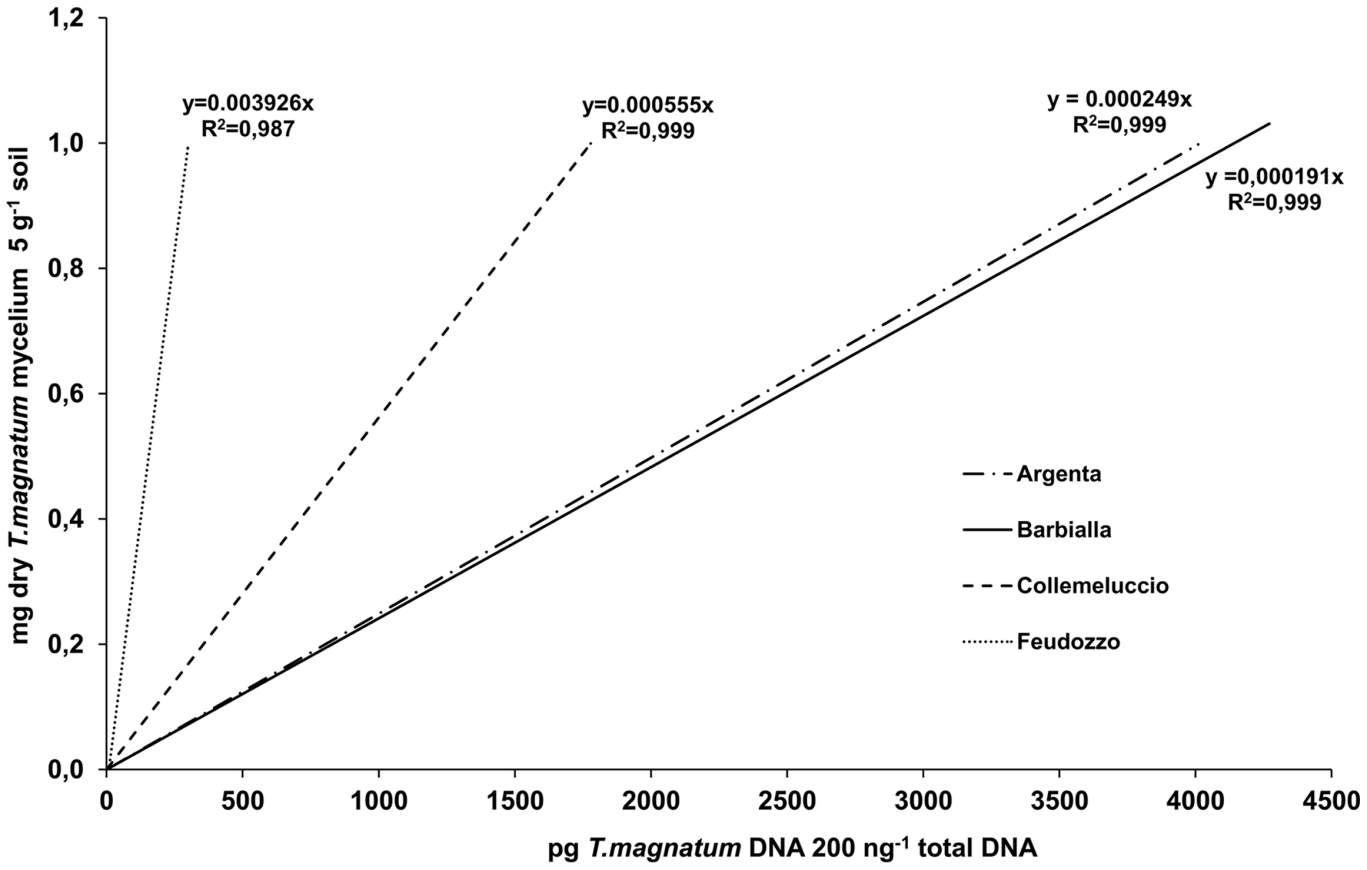
Soil-specific calibration curves for the absolute quantification of *T. magnatum* mycelial biomass. Linear curves were obtained by plotting the log of dry weight of fungal biomass (mg g^−1^ of dried soil) against the *T. magnatum* DNA concentration (pg 200 ng^−1^ of total DNA).

Calibration curves generated for each soil type and used to convert *T. magnatum* DNA concentration data to mycelium biomass are shown in Fig. 1. Slopes were calculated including qPCR data from all *T. magnatum* serial dilutions except those containing 2*10^−6^ mg of gleba per g of soil, because of the inconsistent and variable results obtained with these dilutions. No fluorescence signals were detected after 40 cycles in any *T. magnatum* mycelium-free replicate of each truffle ground. Similar curves were obtained from Argenta and Barbialla soils but their slopes strongly diverged from those of Collemeluccio and Feudozzo.

### Spatial distribution

*T. magnatum* mycelium was detected in all soil samples taken in correspondence of the fruiting points (P0). On average, dry mycelial biomass in P0 samples ranged from 3.93 µg g^−1^ of soil (Barbialla) to 6.87 µg g^−1^ of soil (Feudozzo), but no statistical differences (*p* = 0.88) were found among the different experimental sites. No correlation was found between the weight of *T. magnatum* ascomata and the amount of its extra-radical mycelium in P0 samples (r = 0.029, *p* = 0.86).

**Figure 2:**
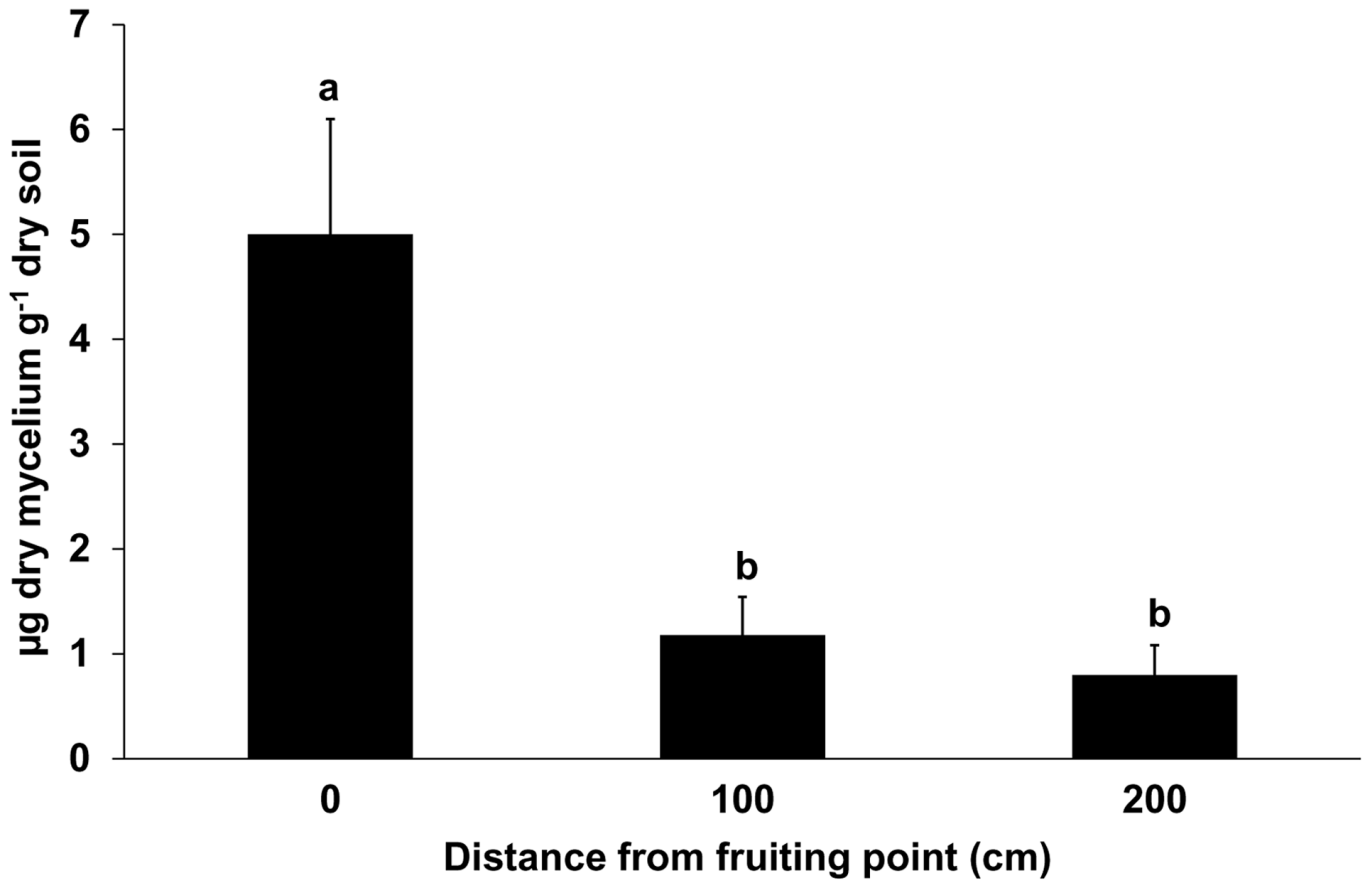
Mean amount of extra-radical soil mycelium of *T. magnatum* at progressive distances from the fruiting point. Error bars represent standard error (n = 49). Statistical analysis was carried out on log-transformed values [y = log(x+1)]. Different letters indicate significant differences between sampling points (*p*<0.0001).

In contrast with the data from P0, 10% and 22% of the soils sampled at 100 cm (P1) and 200 cm (P2) from the fruiting points, respectively, gave no detectable amount of *T. magnatum* mycelium. The highest and lowest percentages of samples lacking in the target mycelium were from Feudozzo (18% for P1 and 33% for P2 samples) and Barbialla (0% for P1 and 8% for P2 samples). BIC analysis revealed that the model including only the sampling position provided the best fit whereas ANOVA showed that this variable affected *T. magnatum* mycelium in soil (*p*<0.0001). A significantly higher presence of *T. magnatum* mycelium was revealed in samples P0 than both P1 and P2 (*p*<0.0001) whereas non statistical differences were found between P1 and P2 samples (*p* = 0.63) (Fig. 2). However, although the quantity of mycelium significantly decreased going farther away from P0, exceptions to this rule were found when two *T. magnatum* ascomata were collected in the same fruiting season within less than 2 m from each other. In some cases, the overlapping of the sampling spots resulted in slightly higher amounts of *T. magnatum* mycelium in P1 or P2 than P0 samples (see S3 Fig.).

### Seasonal dynamics

**Figure 3:**
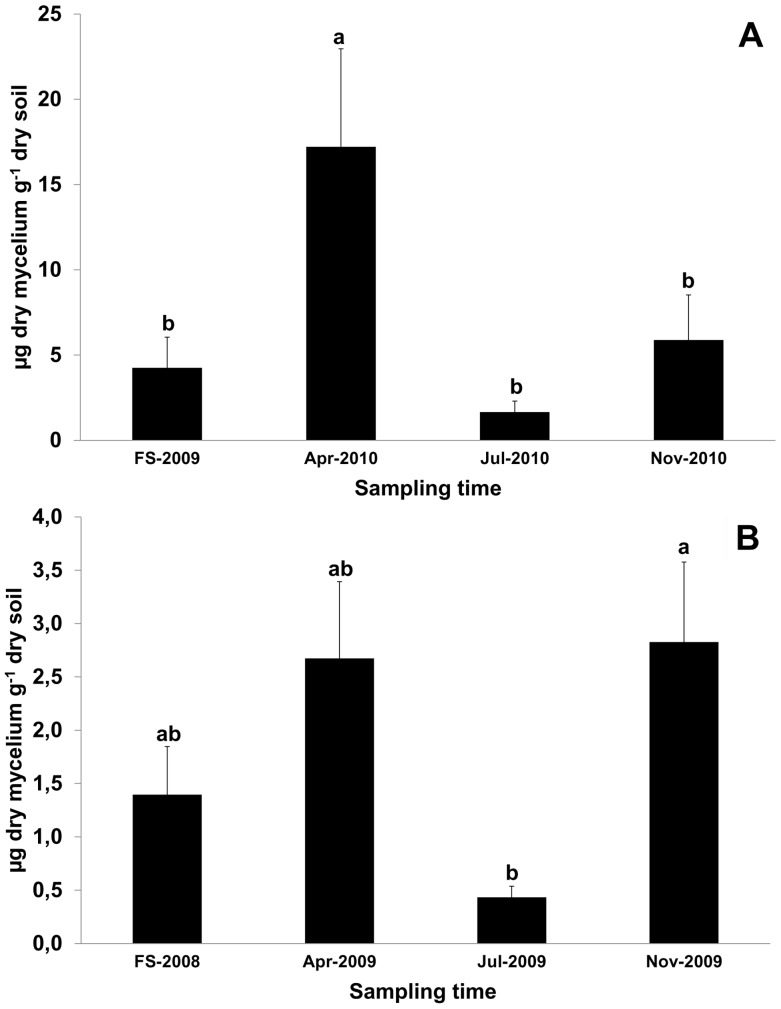
Mean amount of extra-radical soil mycelium of *T. magnatum* across four consecutive sampling periods. Data are referred to Feudozzo (A) and Argenta (B) truffle grounds. FS  =  fruiting season: from 3-Nov-2009 to 3-Dec-2009 (A) and from 27-Oct-2008 to 18-Nov-2008 (B). Different scales are used on the y-axis because of the different range of values gathered from Argenta and Feudozzo. Error bars represent standard error (n = 21, 7 fruiting points ×3 sampling distances for each truffle grounds). Statistical analysis was carried out on log-transformed values [y = log(x+1)]. Different letters indicate significant differences between sampling periods (*p*<0.01 in A and *p*<0.05 in B).

**Figure 4:**
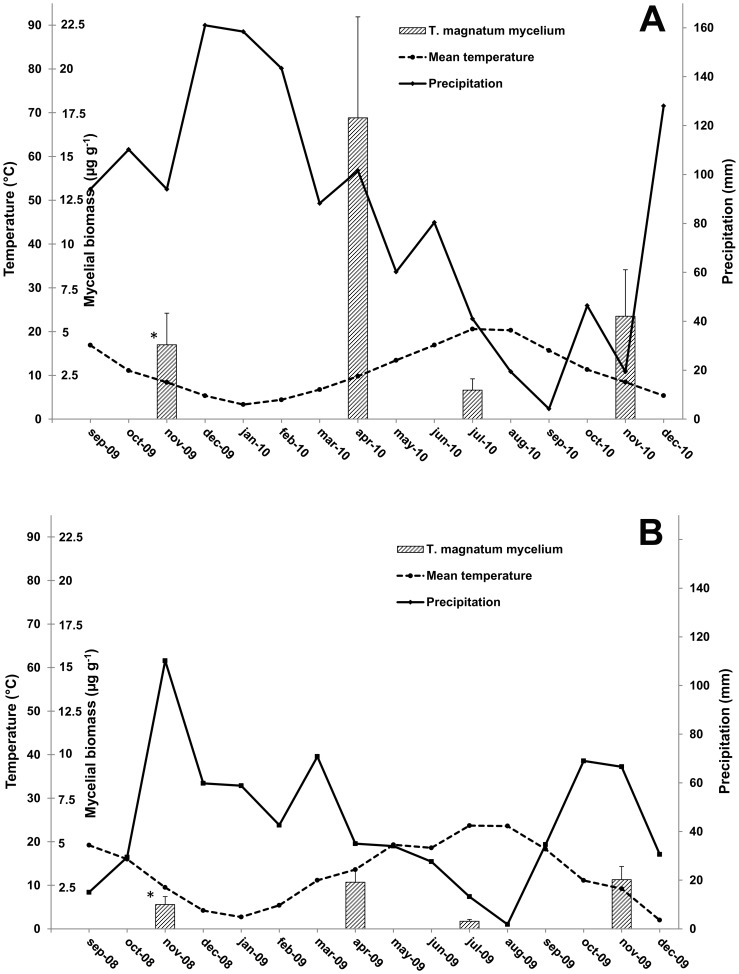
Bagnouls-Gaussen diagrams of Feudozzo (A) and Argenta (B) truffle grounds. Climate data span from September 2009 to December 2010 (A) and from September 2008 to December 2009 (B). Mean amounts of extra-radical soil mycelium of *T. magnatum* (µg g^−1^ of dried soil) obtained from seasonal dynamic analyses are added as columns in correspondence of the respective sampling month to facilitate the comparison between climate and mycelial biomass data (error bars represent standard error). *  =  Fruiting season: from 3-Nov-2009 to 3-Dec-2009 (A) and from 27-Oct-2008 to 18-Nov-2008 (B).

Real-time data obtained from the samples collected in April, July and November at Feudozzo (2010) and Argenta (2009) were analysed together with those from the respective productive spots sampled through the previous fruiting season. *T. magnatum* was detected in 93% of all soil samples. The 7% of samples where *T. magnatum* was not detected, were collected in July (n = 3), November (n = 4) and during the fruiting season (n = 4). BIC analysis revealed that the model including only the sampling season variable provided the best fit. Sampling season was found to affect *T. magnatum* mycelium at both experimental sites (*p*<0.05). At Feudozzo, the amount of *T. magnatum* mycelial biomass detected in April 2010 samples (17.2 µg g^−1^ on average) was significantly higher (*p*<0.01) than those obtained for the other sampling times (Fig. 3a), whereas at Argenta, significant differences (*p*<0.05) were found between samples collected in November 2009 and in July 2009 (Fig. 3b). In this latter site, a high mean amount of *T. magnatum* mycelial biomass was also obtained from April 2009 samples (2.6 µg g^−1^) but differences between April 2009 and July 2009 samples were notable but not significant (*p* = 0.053). The amount of *T. magnatum* mycelium decreased strongly in July, in particular at Argenta where it was found to be 0.4 µg g^−1^ (Fig. 3b). In both truffle grounds, July sampling was the only one to be carried out during the dry season as shown by the Bagnouls-Gaussen diagrams (Fig. 4a and 4b).

## Discussion

This work is one of the first attempts to evaluate the distribution of *T. magnatum* mycelium in soil. Previously, Zampieri *et al.* [15] assessed *T. magnatum* mycelium in a natural truffle-ground by a qualitative nested PCR approach. However, spatial and seasonal variation of *T. magnatum* in soil by qPCR has never been previously assessed.

This molecular technique is a powerful tool to estimate soil fungal biomass of a target species, although caution is advised for comparing quantitative data obtained by different methods or from different environments [20], [30]. Wallander *et al.* [33] recognized nucleic acid extraction process and soil type as the main factors that may influence quantitative PCR success. Soil characteristics may affect not only the yield of extracted DNA but also the community composition and the fungi-to-bacteria ratio, particularly when single DNA extractions are performed for each soil sample [34]. In the present study we reduced these biases by processing all soil samples with the same experimental conditions and generating soil-specific calibration curves for each truffle ground. The effects of the soil properties on the success of *T. magnatum* DNA extraction and its amplification in qPCR were also confirmed in this study. In fact, the experimental sites with similar soil properties (Argenta and Barbialla) gave similar calibration curves, having very different slopes from those obtained for Feudozzo and Collemeluccio soils which differ in texture (higher clay content), pH and organic matter content. Moreover, the use of these soil-specific calibration curves made it possible to exclude the experimental site as a variable affecting the concentration of *T. magnatum* extra-radical mycelium in soil.

The mean amount of extra-radical mycelium obtained from *T. magnatum* fruiting points (4 to 7 µg g^−1^ of soil, dry weight/dry weight) differs from those calculated for other ECM species. Slightly higher values were from *Boletus edulis* Bull. (20 µg g^−1^ of soil) and *Lactarius deliciosus* (L.) Gray (33 to 153 µg g^−1^ of soil) productive patches, during the fruiting season [35], although these differences might be due to the different experimental procedures (i.e. fresh rather than dry mycelium used to create standard curves). Markedly higher values were found for *T. aestivum* (2.18 mg g^−1^ of soil) and *T. melanosporum* (0.2–0.4 mg g^−1^ of soil) from spring samplings in productive truffle orchards [26]–[27]. However, irrespective of the absolute values obtained by quantitative PCR assays, a positive correlation between extra-radical mycelium and ectomycorrhizas was found for *L. deliciosus*, *Rhizopogon* spp., and *B. edulis* [24], [36]. Ectomycorrhizas of many ECM species are also commonly found beneath their fruiting bodies [37]–[41]. On the contrary, not even one *T. magnatum* ECM tip was found beneath the fruiting points under investigation [10], regardless of the amount of extra-radical mycelium detected by qPCR. Indeed, the ecological strategy of *T. magnatum* differs from that of the other truffles whose ectomycorrhizas tend to dominate the ECM community in the upper soil layers (10–30 cm) of the fruiting patches [41]–[44].

The fruiting pattern of *T. magnatum* in forest landscape indicates a patchy distribution of this species although it forms larger mycelial patches with respect to what is expected from fruiting sites [15], [28]. Our analysis also showed a high heterogeneity in spatial and temporal distribution of *T. magnatum* extra-radical mycelium within each patch. This growing pattern, common to soil-born fungi, results from the interaction of numerous biotic and abiotic drivers of the soil fungal communities also at very fine-scale [42], [45]–[48]. However, throughout the fruiting season, the presence of a gradient of *T. magnatum* extra-radical mycelium in soil has been proved, with the highest concentration of mycelial biomass in the soil surrounding the fruiting body and a decreasing trend going away from this point. Only the presence of a cluster of fruiting points might alter this trend. The relationship between the spatial distribution of the belowground mycelial system and fruiting bodies in ECM fungi is conflicting. Similarly to *T. magnatum*, Guidot *et al.* [47] found that below-ground biomass of *Hebeloma cylindrosporum* Romagn. decreased with increased distance from the fruiting bodies and no DNA of this species was detected in soil samples collected at more than 50 cm away. On the contrary, in stipitate hydnoid fungi the highest amounts of mycelium often do not fit with the fruiting position [49]. This different distribution pattern of mycelium in soil could explain the positive correlation between fruiting body productivity and extra-radical mycelium found for *T. magnatum* [28] but not for other ECM mushroom species [24].

During the year, *T. magnatum* mycelium inside the productive patches undergoes fluctuations only depending on the season whereas the fruiting position no longer affects the mycelial distribution after the fruiting season. In early spring, the mean quantity of mycelium tends to increase and redistributes within the fruiting patches. In July, the quantity of *T. magnatum* mycelium in soil, probably due to the high temperatures and/or the scarce rainfall characterizing the climate of *T. magnatum* production areas [50], significantly decreases whereas in the following autumn it tends to increase and concentrate again at the future fruiting points. The evident differences in mean mycelial biomass obtained from the analyses of seasonal dynamic at Feudozzo and Argenta are also probably due to the different climate trend in the two truffle grounds over the sampling years. The lower mean and max temperatures (in particular those of the hottest month), the higher rainfall and number of rainy days as well as the shorter dry period (two *vs* four months, see Fig. 4) registered at Feudozzo in 2010 could explain the higher amounts of *T. magnatum* mycelium than those obtained from Argenta in 2009. This different climate trend might also be the cause of gap in truffle production between Feudozzo (11 fruiting bodies in 2010) and Argenta (only 2 fruiting bodies in 2009). On the basis of these preliminary analyses, it seems that *T. magnatum* is sensitive to the summer precipitations and temperatures as proved by Büntgen *et al.* [51] for the black truffle species *T. melanosporum* and *T. aestivum*. From this point of view, *T. magnatum* might be more affected by the climatic changes because of its more stringent ecological requirements.

Slightly in contrast with our results, Zampieri *et al.* [15] found a decrease of *T. magnatum* mycelium in spring. However, these authors applied a different approach to evaluate *T. magnatum* (qualitative *vs* quantitative PCR) and carried out the soil sampling in different months (May *vs* April). On the other hand, an increase of *T. magnatum* mycelial biomass in soil during spring is not surprising because it has been demonstrated that the seasonal dynamic of the ECM mycelia is mainly dependent on plant photosynthate availability and on the fine root growth [52]–[53], which mainly occur during this season in Mediterranean areas [54]. However, a similar study on the seasonal dynamic of the mycelium of *B. edulis* and *L. delicious* showed that the mycelium fluctuations are related to the climatic conditions but they also seem to depend on the ECM taxon [35]. In fact, *B. edulis* soil mycelium showed a higher concentration in February-March samplings, whereas the concentration of *L. delicious* has two peaks, the larger one in December and the smaller one in March. Differences between these two ECM fungi were attributed to their different hyphal exploration type. However, the physiology of individual taxa, and in particular the extent of mycorrhizal saprotrophy, also has an important role determining their seasonal mycelium fluctuation [55]. As hypothesized for the maturation process of *T. magnatum* ascomata [56], microbial community composition might also be crucial for the mycelial dynamics of this truffle species.

These results provide useful suggestions for evaluating the presence and distribution of *T. magnatum* in a truffle ground. For this purpose, the sampling strategy has to be adapted to the aim of the survey. Early spring sampling seems more appropriate to evaluate the health status of a truffle ground because climatic conditions are usually more suitable for *T. magnatum* mycelium growth in soil and the mycelium is more uniformly distributed in the fruiting patches. In contrast, autumn samplings are more appropriate for studying fruiting events. This study also gives new insights on *T. magnatum* ecology although the relationships between *T. magnatum* mycelial growth and climatic soil parameters have to be investigated in depth. However, as for the black truffle species [57], the preliminary results from this study highlight the need to test cultural practices improving summer thermo-hydric balance in natural *T. magnatum* areas to safeguard the presence of its mycelium in the soil and to promote its fructification.

## Supporting Information

S1 Fig **Map of Italy with the location of the four experimental sites.**
(JPG)

S2 Fig **Mean standard curve resulting from 28 independent qPCR runs.** This curve was generated by plotting the means of the Ct values from qPCR runs against the logarithm of a known quantity of *T. magnatum* genomic DNA. Variability is shown as the mean Ct value ± SD.
(DOC)

S3 Fig **Sampling scheme and** ***T. magnatum*** **mycelial biomass of two overlapping fruiting spots.** The related ascomata were collected about 110 cm away from each other, during autumn 2009 in Barbialla truffle ground. The collection date (CD) is reported for each ascoma. Different circles (black, grey and white) correspond to different sampling position (P0, P1 and P2) and dotted lines indicate the sampling directions within each fruiting spot.
(DOC)

S1 Table **List of ECM and non-ECM tree and shrub species of the four experimental sites.** Vegetation surveys were carried out using the Braun-Blanquet methodology (1964). Percent cover of the different plant species in the different tree and shrub strata was estimated as a percentage of the surface (values <5% are reported as +). ECM trees growing in *T. magnatum* productive patches are indicated as ^a^ (dominant) or ^b^ (sporadic).
(DOC)

S1 File **Datasets used for statistical analyses.**
(XLSX)
