# Supplementary figures and images for: Spatio-Temporal Dynamic of Tuber magnatum Mycelium in Natural Truffle Grounds
Source: PLoS One. 2014 Dec 23;9(12):e115921. doi: 10.1371/journal.pone.0115921 (PMC4275250; doi:10.1371/journal.pone.0115921)

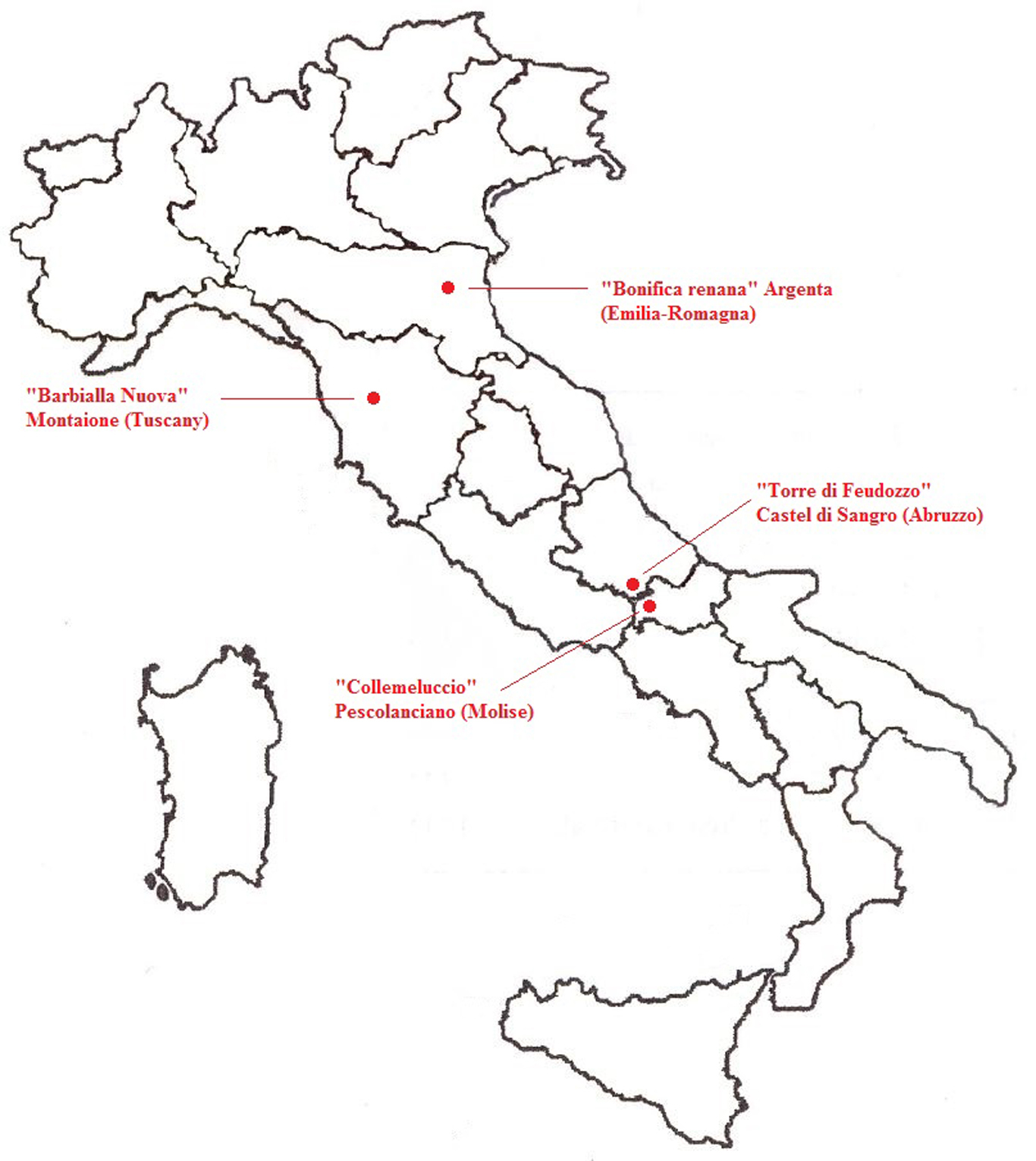

Supplement: S1 Fig — Map of Italy with the location of the four experimental sites. (JPG) [file pone.0115921.s001.jpg]
