# Supplementary material for: Spatio-Temporal Dynamic of Tuber magnatum Mycelium in Natural Truffle Grounds
Source: PLoS One. 2014 Dec 23;9(12):e115921. doi: 10.1371/journal.pone.0115921 (PMC4275250; doi:10.1371/journal.pone.0115921)
Supplement: S2 Fig — Mean standard curve resulting from 28 independent qPCR runs. This curve was generated by plotting the means of the Ct values from qPCR runs against the logarithm of a known quantity of T. magnatum genomic DNA. Variability is shown as the mean Ct value ± SD. (DOC) [file pone.0115921.s002.doc]

**Figure S2. Mean standard curve resulting from 28 independent qPCR runs.** This curve was generated by plotting the means of the Ct values from qPCR runs against the logarithm of a known quantity of *Tuber magnatum* genomic DNA. Variability is shown as the mean Ct value ± SD.
