# Supplementary material for: Spatio-Temporal Dynamic of Tuber magnatum Mycelium in Natural Truffle Grounds
Source: PLoS One. 2014 Dec 23;9(12):e115921. doi: 10.1371/journal.pone.0115921 (PMC4275250; doi:10.1371/journal.pone.0115921)
Supplement: S3 Fig — Sampling scheme and T. magnatum mycelial biomass of two overlapping fruiting spots. The related ascomata were collected about 110 cm away from each other, during autumn 2009 in Barbialla truffle ground. The collection date (CD) is reported for each ascoma. Different circles (black, grey and white) correspond to different sampling position (P0, P1 and P2) and dotted lines indicate the sampling directions within each fruiting spot. (DOC) [file pone.0115921.s003.doc]

**Figure S3. Sampling scheme and *Tuber magnatum* mycelial biomass (μg of dry mycelium g^-1^ of dried soil) of two overlapping fruiting spots.** The related ascomata were collected about 110 cm away from each other, during Autumn 2009 in Barbialla truffle ground. The collection date (CD) is reported for each ascoma. Different circles (black, grey and white) correspond to different sampling position (P0, P1 and P2) and dotted lines indicate the sampling directions within each fruiting spot.
