# Supplementary material for: Spatio-Temporal Dynamic of Tuber magnatum Mycelium in Natural Truffle Grounds
Source: PLoS One. 2014 Dec 23;9(12):e115921. doi: 10.1371/journal.pone.0115921 (PMC4275250; doi:10.1371/journal.pone.0115921)
Supplement: S1 Table — List of ECM and non-ECM tree and shrub species of the four experimental sites. Vegetation surveys were carried out using the Braun-Blanquet methodology (1964). Percent cover of the different plant species in the different tree and shrub strata was estimated as a percentage of the surface (values <5% are reported as +). ECM trees growing in T. magnatum productive patches are indicated as a (dominant) or b (sporadic). (DOC) [file pone.0115921.s004.doc]

**Table S1. List of ECM and non-ECM tree and shrub species of the four experimental sites.** Vegetation surveys were carried out using the Braun-Blanquet methodology (1964). Percent cover of the different plant species in the different tree and shrub strata was estimated as a percentage of the surface (values < 5% are reported as +). ECM trees growing in *T. magnatum* productive patches are indicated as ^a^ (dominant) or ^b^ (sporadic).

| Plant species | ECM | non-ECM | Barbialla (Tuscany) | Argenta (Emilia-Romagna) | Feudozzo (Abruzzo) | Collemeluccio (Molise) |
| --- | --- | --- | --- | --- | --- | --- |
| Tree layer |  |  |  |  |  |  |
| *Abies alba* Miller | * |  |  |  |  | 7^a^ |
| *Acer campestre* L. |  | * | + |  | + | + |
| *Acer opalus* Mill. |  | * |  |  | + |  |
| *Aesculus hippocastanum* L. |  | * |  | + |  |  |
| *Albizzia julibrissin* (Willd.) Durazzo |  | * |  | + |  |  |
| *Carpinus betulus* L. | * |  |  |  | 15 | + |
| *Cedrus atlantica* Man. | * |  |  | 5 |  |  |
| *Celtis australis* L. |  | * |  | + |  |  |
| *Cercis siliquastrum* L. |  | * |  | + |  |  |
| *Corylus avellana* L. | * |  |  |  |  | +^b^ |
| *Crataegus monogyna* Jacq. |  | * | + |  |  | + |
| *Fraxinus excelsior* L. |  | * |  |  | + |  |
| *Fraxinus ornus* L. |  | * | + |  | + |  |
| *Hedera helix* L. |  | * | + | + | + |  |
| *Magnolia grandiflora* L. |  | * |  | + |  |  |
| *Malus sylvestris* (L.) Mill. |  | * |  |  |  | + |
| *Ostrya carpinifolia* Scop. | * |  | 48^a^ |  |  | + |
| *Pinus nigra* Arnold | * |  |  | + |  |  |
| *Pinus pinaster* Aiton | * |  |  | + |  |  |
| *Pyrus pyraster* Burgsd. |  | * |  |  |  | 7 |
| *Platanus occidentalis* L. |  | * |  | 12 |  |  |
| *Populus alba* L. | * |  | 14^a^ |  |  |  |
| *Populus canadensis* Moench | * |  |  |  |  | 7^b^ |
| *Populus nigra* L. | * |  |  | 12^a^ |  | + |
| *Populus tremula* L. | * |  |  |  | +^b^ |  |
| *Quercus cerris* L. | * |  | +^b^ |  | 32^a^ | 46^a^ |
| *Quercus ilex* L. | * |  | 5^b^ |  |  |  |
| *Quercus petraea* (Mattuschka) Liebl. | * |  | 6 |  |  |  |
| *Quercus pubescens* Willd. | * |  | + |  |  |  |
| *Quercus robur* L. | * |  |  | + |  |  |
| *Sophora japonica* L. |  | * |  | 9 |  |  |
| *Sorbus torminalis* (L.) Crantz |  | * |  |  |  | + |
| *Taxodium distichum* (L.) Richard |  | * |  | + |  |  |
| *Tilia platyphyllos* Scop. | * |  |  |  |  | + |
| *Tilia vulgaris* Hayne | * |  |  | 14^a^ |  |  |
|  |  |  |  |  |  |  |
| Shrub layer |  |  |  |  |  |  |
| *Abies alba* Miller | * |  |  |  |  | 18^a^ |
| *Acer campestre* L. |  | * | + |  | + | + |
| *Acer opalus* Mill. |  | * |  |  | + |  |
| *Acer pseudoplatanus* L. |  | * |  |  | + |  |
| *Buxus sempervirens* L. |  | * |  | + |  |  |
| *Carpinus betulus* L. | * |  |  |  | 11^b^ |  |
| *Carpinus orientalis* Mill. | * |  |  |  | + |  |
| *Clematis vitalba* L. |  | * | + |  | 5 |  |
| *Cornus mas* L. |  | * |  |  | + |  |
| *Cornus sanguinea* L. |  | * | 8 | + | 16 | + |
| *Coronilla emerus* L. |  | * | + |  |  |  |
| *Corylus avellana* L. | * |  |  |  | +^b^ | +^b^ |
| *Crataegus laevigata* (Poir.) DC. |  | * |  |  | + |  |
| *Crataegus monogyna* Jacq. |  | * | + |  | + | + |
| *Daphne laureola* L. |  | * |  |  | + |  |
| *Deutzia japonica* Hort. |  | * |  | + |  |  |
| *Euonymus europaeus* L. |  | * |  |  | + | + |
| *Ficus carica* L. |  | * | + |  |  |  |
| *Fraxinus excelsior* L. |  | * |  |  | + |  |
| *Fraxinus ornus* L. |  | * | + |  | + |  |
| *Hedera helix* L. |  | * | + | + | + |  |
| *Juniperus communis* L. |  | * |  |  |  | + |
| *Laburnum anagyroides* Medik. |  | * |  |  | + | + |
| *Laurus nobilis* L. |  | * | + |  |  |  |
| *Ligusticum lucidum* Miller |  | * |  | + |  |  |
| *Ligustrum vulgare* L. |  | * | + |  | 16 | + |
| *Lonicera caprifolium* L. |  | * | + |  |  |  |
| *Malus sylvestris* (L.) Mill. |  | * |  |  | + |  |
| *Melissa officinalis* L. |  | * |  | + |  |  |
| *Ostrya carpinifolia* Scop. | * |  | +^a^ | + | + |  |
| *Populus alba* L. | * |  | +^a^ |  |  |  |
| *Populus nigra* L. | * |  |  |  |  | + |
| *Populus tremula* L. | * |  |  |  | +^b^ |  |
| *Prunus avium* L. |  | * |  |  | + |  |
| *Prunus cerasifera* Ehrh. |  | * |  |  | + |  |
| *Prunus laurocerasus* L. |  | * |  | + |  |  |
| *Prunus spinosa* L. |  | * | + |  | + | + |
| *Pyracantha coccinea* M. J. Roemer |  | * | + |  |  |  |
| *Pyrus pyraster* Burgsd. |  | * |  |  | + | + |
| *Quercus cerris* L. | * |  | +^b^ |  | 6^a^ | +^a^ |
| *Quercus ilex* L. | * |  | +^b^ |  |  |  |
| *Quercus pubescens* Willd. | * |  | + |  |  |  |
| *Robinia pseudoacacia* L. |  | * |  | + |  |  |
| *Rosa arvensis* Hudson |  | * | + |  | 5 | + |
| *Rosa canina* L. sensu Bouleng. |  | * | + |  |  |  |
| *Rosa sempervirens* L. |  | * | + |  |  |  |
| *Rubus canescens* DC. |  | * | 18 |  |  |  |
| *Rubus hirtus* Waldst. & Kit. |  | * |  |  | 38 | + |
| *Rubus idaeus* L. |  | * | + |  |  |  |
| *Rubus ulmifolius* Schott |  | * |  |  | + |  |
| *Salix alba* L. | * |  |  |  | + |  |
| *Salix apennina* A.K. Skvortsov | * |  |  |  | + |  |
| *Salix caprea* L. | * |  |  |  | 8^b^ |  |
| *Salix purpurea* L. | * |  |  |  | + |  |
| *Sambucus ebulus* L. |  | * | + |  |  |  |
| *Sambucus nigra* L. |  | * |  | + | + |  |
| *Sorbus domestica* L. |  | * | + |  |  |  |
| *Syringa vulgaris* L. |  | * |  | + |  |  |
| *Tamus communis* L. |  | * |  |  | + |  |
| *Tilia platyphyllos* Scop. | * |  |  |  | + |  |
| *Ulmus minor* Miller |  | * | + |  | + |  |

Reference

Braun-Blanquet, J. (1964). Pflanzensoziologie: grundzüge der vegetationskunde. Zweite, umgearbeitete und vermehrte Auflage. Springer-Verlag: Wien. 865 pp.
